# Safety Characterization and Antimicrobial Properties of Kefir-Isolated *Lactobacillus kefiri*

**DOI:** 10.1155/2014/208974

**Published:** 2014-05-13

**Authors:** Paula Carasi, Mariángeles Díaz, Silvia M. Racedo, Graciela De Antoni, María C. Urdaci, María de los Angeles Serradell

**Affiliations:** ^1^Cátedra de Microbiología, Departamento de Ciencias Biológicas, de La Plata, 47 y 115 s/n, CP, 1900 La Plata, Argentina; ^2^Laboratoire de Microbiologie et Biochimie Appliquée (LBMA), Université de Bordeaux, UMR 5248, Bordeaux Sciences Agro, 1 Cours du Général de Gaulle, 33175 Gradignan, France

## Abstract

Lactobacilli are generally regarded as safe; however, certain strains have been associated with cases of infection. Our workgroup has already assessed many functional properties of * Lactobacillus kefiri*, but parameters regarding safety must be studied before calling them probiotics. In this work, safety aspects and antimicrobial activity of * L. kefiri* strains were studied. None of the * L. kefiri* strains tested caused *α*- or *β*-hemolysis. All the strains were susceptible to tetracycline, clindamycin, streptomycin, ampicillin, erythromycin, kanamycin, and gentamicin; meanwhile, two strains were resistant to chloramphenicol. On the other hand, all *L. kefiri* strains were able to inhibit both Gram(+) and Gram(−) pathogens. Regarding the *in vitro* results, *L. kefiri* CIDCA 8348 was selected to perform *in vivo* studies. Mice treated daily with an oral dose of 10^8^ CFU during 21 days showed no signs of pain, lethargy, dehydration, or diarrhea, and the histological studies were consistent with those findings. Moreover, no differences in proinflammatory cytokines secretion were observed between treated and control mice. No translocation of microorganisms to blood, spleen, or liver was observed. Regarding these findings, *L. kefiri* CIDCA 8348 is a microorganism isolated from a dairy product with a great potential as probiotic for human or animal use.

## 1. Introduction

Kefir grains are composed of a complex community of yeasts, lactic acid, and acetic acid bacteria confined in a matrix of polysaccharides and proteins [1]. The product obtained by fermentation of milk using these grains is called “kefir” and several health-promoting properties have been associated to its consumption [2–5].

As it is known, probiotics are “live microorganisms which, administered in adequate amounts, exert a beneficial effect to the health of the host” [6]. Specific strains of lactic acid bacteria, in particular some of the genera* Lactobacillus*, are extensively used as probiotics [7, 8] since their ability to modulate the immune system has been demonstrated [9, 10] as well as their capacity to inhibit the growth or invasion of pathogenic bacteria and parasites [11–13].

The study of the beneficial properties attributed to isolated microorganisms constitutes a field of great interest for the development of functional foods. Lactobacilli are generally regarded as safe (GRAS) and most of them (as* Lactobacillus kefiri*) are included in the QPS list of the European Union [14] due to their long history of use in fermented dairy products and their presence in human intestinal tract. However, certain* Lactobacillus* strains have been associated with cases of sepsis, endocarditis, or bacteremia, mostly in association with a severe underlying disease [15–18]. On the other hand, the absence of the acquired antimicrobial resistance is a very important criterion for evaluating the safety of lactic acid bacteria (LAB) used as food started or probiotics [19]. The breakpoints for the antibiotic list were defined by the European Food Safety Authority (EFSA) in order to assess the bacterial resistance to antibiotics of human or veterinary importance [20, 21].

Our workgroup has isolated and characterized numerous species of LAB and yeasts from kefir, including several strains of* Lactobacillus kefiri* [22–24], one of the most predominant species present in kefir-fermented milk (ranged from 2 × 10^8^ to 1 × 10^9^ *L. kefiri* cells mL^−1^) [25].

We have already demonstrated the potential of* L. kefiri* as a probiotic microorganism* in vitro* after verifying that secretion products and surface proteins from these heterofermentative lactobacilli exert a protective action against the invasion of* Salmonella enterica* serovar Enteritidis [26] and that they are able to antagonize the cytotoxic effects of clostridial toxins on Vero cells [27]. On the other hand, it has been demonstrated that* L. kefiri* strains are able to preserve a high percentage of viability after both spray-drying [28, 29] and freeze-drying procedures [30]. However, no parameter regarding* L. kefiri's* safety was ever evaluated. Since it is known that both the beneficial properties such as harmful characteristic are dependent on the strain, the individual study of the safety of potential probiotic microorganisms should be considered.

Taking into account the potential of* L. kefiri* as a novel probiotic, we reported in this work some safety characteristics of* L. kefiri* strains, as well as the capacity of strains to produce antimicrobial compounds against some intestinal pathogens.

## 2. Materials and Methods 

### 2.1. Bacterial Strains and Culture Conditions

Pure cultures used in this study comprised* Lactobacillus kefiri* strains CIDCA 8321, 8345, 8348, 83111, 83113, and 83115 [23, 31]. These bacteria were cultured in MRS (Difco, Detroit, USA) for 48 h at 37°C. The following pathogenic bacteria were also used,* Enterococcus faecalis* ATCC 29212,* Staphylococcus aureus* ATCC 6538,* Shigella flexneri* ATCC 9199,* Pseudomona aeruginosa* ATCC 15442, a clinical isolate of* Salmonella enterica* serovar Enteritidis CIDCA 101 (Hospital de Pediatría Professor Juan P. Garrahan, Buenos Aires, Argentina), enterohemorrhagic* Escherichia coli* EDL 933,* Listeria monocytogenes* ATCC 7644, and* Bacillus cereus* ATCC 10876. All mentioned strains, except* B. cereus*, were grown using brain heart infusion (BHI) broth (Biokar Diagnostics, Beauvais, France) in agitation at 37°C for 16 h.* B. cereus* was grown in BHI growth supplemented with dextrose (Anedra, Argentina) 1 g L^−1^ (BHIg) in agitation at 37°C for 16 h.

### 2.2. Hemolysis

Hemolysis was tested by growth of the strains on LAPTg agar (peptone 15 g L^−1^; tryptone 10 g L^−1^; dextrose 10 g L^−1^; yeast extract 10 g L^−1^; Tween 80 1 g L^−1^; and bacteriological agar 15 g L^−1^) supplemented with 5% human blood (group O) and incubated for 48 h at 37°C under aerobic conditions. The appearance of clear zones around the bacterial colonies indicated the presence of *β*-hemolysis whereas green zones around the colonies suggested *α*-hemolysis.* Enterococcus faecalis* ATCC 29212 was included as a positive hemolytic control.

### 2.3. Minimum Inhibitory Concentration (MIC) for Antibiotic Resistance

The minimum inhibitory concentrations (MICs) of the antimicrobial agents tested (Table 1) were determined by broth microdilution according to the ISO 10932/IDF 233 standard from 2010 [32]. All antibiotics (Sigma-Aldrich, USA) were dissolved for preparing stock solutions of 1280 *μ*g mL^−1^. Stock solutions were diluted in LSM broth (90% IST plus 10% MRS) to obtain solutions with preliminary concentrations in the range of 0.25–128 *μ*g mL^−1^. Bacterial inocula were prepared by suspending colonies from 48 h incubated in MRS medium to 5 mL 0.85% NaCl solution. Subsequently, inocula were adjusted to OD_625 nm_ 0.18–0.24 and diluted 1 : 500 in LSM broth for inoculation of microdilution plates by adding 50 *μ*L of diluted inoculum to each well containing 50 *μ*L of an antibiotic solution. In these conditions, the bacterial inoculum was around 2-3 × 10^5^ CFU mL^−1^ in the wells. After incubating plates under anaerobic conditions at 37°C for 48 hours, the MICs value was read as the lowest concentration of an antimicrobial agent in which visible growth was inhibited.

MICs results were compared with the recommended breakpoints for heterofermentative lactobacilli by the EFSA Panel on Additives and Products or Substances used in Animal Feed [20].

### 2.4. PCR Detection of Chloramphenicol Resistance Gene

*Cat*, chloramphenicol acetyltransferase gene, was assessed using the primers and PCR conditions described by Hummel et al. [33]. A plasmid from* L. reuteri* G4 was used as a positive control.

### 2.5. Growth Inhibition of Bacterial Pathogens

The agar spot test described by Schillinger and Lücke [34] was used. Briefly, 5 *μ*L of a suspension OD_625 nm_ 1 of* L. kefiri* strains was spotted into MRS agar and incubated for 24 h at 37°C. The following day, pathogens were seeded into soft BHI agar and plated over the spotted lactobacilli. After 18 h of incubation at 37°C, the inhibition halos were measured. The width of the clear zone (*R*) was calculated as follows: *R* = (dInhib − dSpot)/2, where dInhib is the diameter of the zone without pathogen growth and dSpot is the diameter of the spot. Inhibition scores are as follows: negative (−), *R* < 2 mm; low inhibition capacity (+), 2 mm < *R* < 5 mm; and high inhibition capacity (++), *R* > 6 mm. At least three independent experiments were performed.

### 2.6. *In Vivo* Studies

#### 2.6.1. Ethics Statement

All animal procedures were performed in strict accordance with the guidelines issued by the European Economic Community “86/609.”

#### 2.6.2. Experimental* In Vivo* Protocol

Male 6-week-old Swiss albino mice (Janvier, Le Genest Isle, France) were quarantined 2 weeks after arrival and then randomized by body weight into experimental and control groups of 5–7 animals each. Mice were housed under standard laboratory conditions with free access to food and water. The temperature was kept at 22°C and a 12-hour light/dark schedule was maintained. Mice received by gavage 10^8^ CFU of* L. kefiri* CIDCA 8348 (Lk group) or PBS (control group) daily for 21 days.

#### 2.6.3. Safety Evaluation

Mice were weighted every two days; behavior and signs of pain were analyzed daily [35]. At the end of the experimental protocol, ileum and colon were removed and histological studies were performed using hematoxylin-eosin staining [36].

#### 2.6.4. Translocation Assay

Liver and spleen were removed and blood samples were collected aseptically. Liver and spleen were homogenized in 0.1% sterile PBS (0.1 g of organ per mL) and serially diluted. One hundred microliters of each organ homogenate or blood was plated on VRBG Agar (Biokar Diagnostics, Beauvais, France) for enterobacteria and MRS agar for LAB. Plates were incubated under aerobic conditions for 24 h at 37°C for VRBG and for 48 h at 37°C for MRS before examination.

#### 2.6.5. Microorganism Counts in the Ileum

Ileum content was washed with 1 mL sterile PBS and then serial dilutions were plated as indicated above.

#### 2.6.6. Cytokine Release by Intestine and Colon Explants

Explants were cultured in RPMI medium supplemented with 10% foetal bovine serum (Gibco-Invitrogen, Carlsbad, CA, USA), 10 mg/L streptomycin and 10 IU/mL penicillin G, and 100 mg/L gentamicin (all from Sigma Chemical Co., St. Louis, MO, USA) for 24 h at 37°C in a 5% (v/v) CO_2_-95% (v/v) air atmosphere [37, 38]. Supernatants were collected, centrifuged, and frozen for cytokines (IL-6, IL-17A, TNF-*α*, IFN-*γ*, and GM-CSF) measurements (eBioscience Ready Set Go, France). All assays were performed according to the manufacturer's instructions.

## 3. Results and Discussion

In the present work, six potentially probiotic* L. kefiri* strains isolated from kefir were studied in order to evaluate both their safety and antimicrobial properties.

Since hemolysis is a common virulence factor among pathogens, the first safety parameter evaluated* in vitro* was bacterial hemolytic activity. In this study, none of the* L. kefiri* strains tested caused *α*- or *β*-hemolysis (data not shown). In this genus, hemolytic activity has a very low frequency and only *α*-hemolysis has been reported for lactobacilli isolated from foods and dairy products [39–41].

Another important feature regarding safety is the sensitivity to antibiotics. The results obtained for* L. kefiri* strains are shown in Table 1. All tested bacteria exhibited MIC values lower than the breakpoints recommended for heterofermentative lactobacilli [20] for tetracycline, clindamycin, streptomycin, ampicillin, erythromycin, kanamycin, and gentamicin. However, the strains CIDCA 8321 and 8345 were resistant to chloramphenicol although the amplification of CAT encoding gene was negative for all the* L. kefiri* strains (data not shown). In this regard, Hummel et al. [33] reported that some lactobacilli strains carrying* cat* genes were susceptible to chloramphenicol; meanwhile, in other resistant strains* cat* genes could not be amplified. Further research, such as the study of the distribution of chloramphenicol MICs, could contribute to determine whether resistance is acquired (not acceptable strain) or intrinsic (acceptable strain) according to EFSA [21].

To our knowledge, antibiotic sensitivity of* L. kefiri* was evaluated just in two publications. Nawaz et al. [42] studied one* L. kefiri* strain isolated from a dairy product, which was resistant to kanamycin and tetracycline but sensitive to other antimicrobial agents tested in LSM medium. Chang et al. [43] observed that all the* L. kefiri* strains, among other lactobacilli, isolated from swine intestines were resistant to tetracycline, with MIC values higher than 256 *μ*g mL^−1^, and that they possessed at least one resistance gene. Taking into account that tetracycline is the most widely used antimicrobial agent in swine production, its continuous administration might be selecting tetracycline resistant microorganisms on swine's microbiota. This feature and the different origin of our* L. kefiri* strains could contribute, at least in part, to the disagreement between our results and those from other authors.

The secretion of molecules able to inhibit the growth of pathogens is a desirable characteristic, among others, for a potentially probiotic bacteria [44], and it could also be a technological advantage in the food industry since they might be used as functional starter cultures [45, 46]. We evaluated the pathogen growth inhibition capacity of the six* L. kefiri* strains studied. As observed in Table 2, the inhibition profile was strain dependent, and Gram positive pathogens showed higher sensibility to* L. kefiri* strains than Gram negative bacteria. It is important to notice that the addition of MRS acidified with HCl or lactic acid to pH 4.3 (final pH reached by* L. kefiri* cultures) was not able to produce inhibition of pathogens in our tests (data not shown). All the strains inhibited growth of* Bacillus cereus* and* Staphylococcus aureus* but none of them inhibited enterohemorrhagic* Escherichia coli* (EHEC). The strains* L. kefiri* CIDCA 8321, CIDCA 8348, and CIDCA 83111 were able to inhibit growth of the rest of the tested pathogens. Many mechanisms associated with bacterial inhibition have been described for* Lactobacillus* species [47]. The production of antimicrobial molecules is usually strain dependent, which is in accordance with our results, and the introduction of probiotic bacteria able to inhibit other microorganisms could have a positive impact in animal and human health [48, 49].

Up to here,* L. kefiri* CIDCA 8321, 8348, and 83111 demonstrated to be the most active strains against pathogens; however, CIDCA 8321 showed resistance to chloramphenicol. In consequence, among the other two strains, we selected CIDCA 8348 to perform* in vivo* studies in Swiss mice.

**Figure 1:**
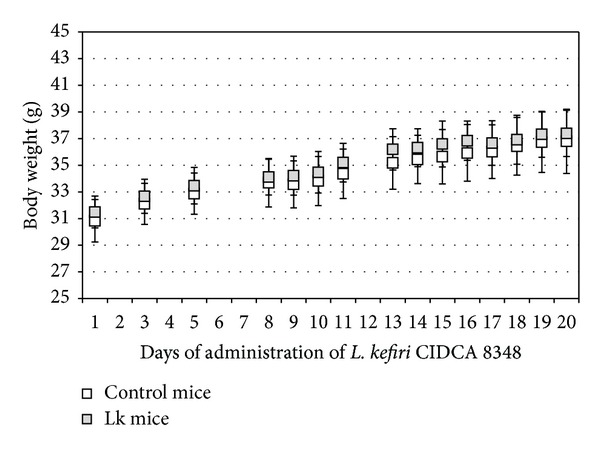
Body weight gain of treated (Lk) and control mice along 21 days of* L. kefir* CIDCA 8348 administration. No differences were observed between control mice and Lk mice (*P* > 0.05).

**Figure 2:**
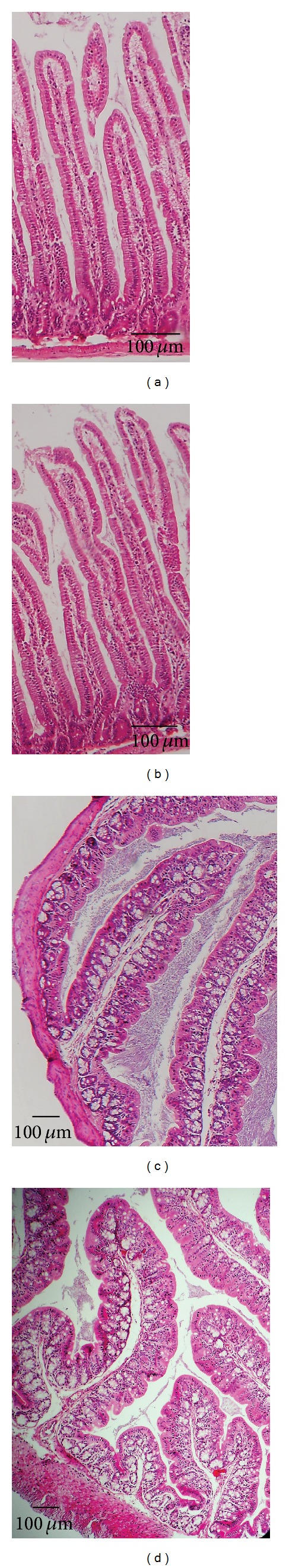
Hematoxylin-eosin staining of ileum and colon section. (a) Ileum of control mice; (b) ileum of mice receiving* L. kefiri* CIDCA 8348 for 21 days; (c) colon of control mice; (d) colon of mice receiving* L. kefiri* CIDCA 8348 for 21 days. No differences were observed among groups in any tissue.

**Figure 3:**
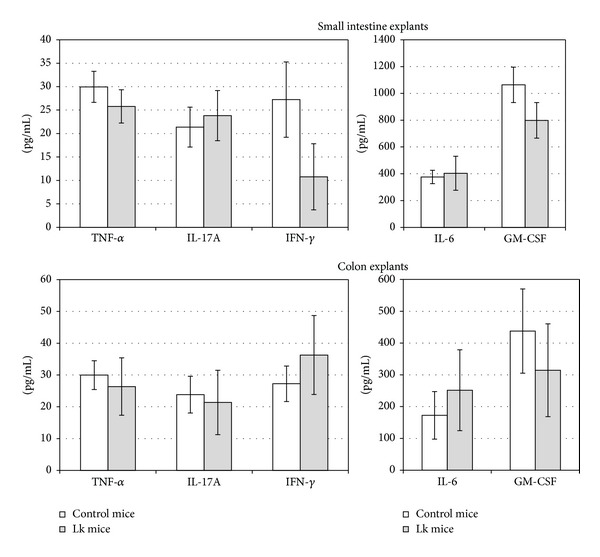
Secretion of proinflammatory cytokines by intestine and colon explants from mice receiving* L. kefiri* CIDCA 8348 for 21 days (Lk) and control mice determined by ELISA. Statistical analysis: one way ANOVA, posttest Bonferroni, *α* = 0.05.

As observed in Figure 1, no differences in body weight were observed between mice that received 100 *μ*L of a 10^9^ CFU mL^−1^ suspension of* L. kefiri* CIDCA 8348 (Lk group) and mice receiving 100 *μ*L of PBS (control group) daily for 21 days. Moreover, there were no differences in food and water intake between groups (data not shown). In accordance with these results, Lk group did not show any signs of pain, lethargy, dehydration, or diarrhea during treatment. No signs of inflammation or damage were observed in any organ during necropsy. Length of each mouse's colon was measured, since it has been reported that increasing levels of inflammation result in shortening of the colon [50]. No significant differences in colon's length of Lk mice and control mice were observed (12.4 ± 0.6 versus 12.6 ± 0.8). Moreover, the histological study of ileum and colon was consistent with the already described observations; no signs of inflammation, edema, erosion/ulceration, crypt loss, or infiltration of mono- and polymorphonuclear cells [51] were observed in Lk mice's tissues (Figure 2), in concordance with previous report by Bolla et al. [30] who administered this strain as a constituent of a mixture of five kefir-isolated microorganisms to BALB/c mice. Additionally, no differences in the secretion levels for proinflammatory cytokines such as IL-6, IL-17A, IFN-*γ*, TNF-*α*, and GM-CSF were observed in the small intestine and colon explants from Lk and control mice (Figure 3). On the other hand, no translocation of microorganisms was observed on blood, spleen, or liver (bacterial counts were negative), which means that the epithelial barrier was not disrupted since intestinal permeability was not affected by* L. kefiri* CIDCA 8348 administration [52]. Besides, the viable counts of enterobacteria (3.5 ± 0.8 × 10^7^ versus 4.8 ± 0.9 × 10^7^) and LAB (1.1 ± 0.6 × 10^7^ versus 2.6 ± 0.8 × 10^7^) in the ileum were comparable between control and treated mice.

## 4. Conclusion

Taking into account all these findings, we conclude that* L. kefiri* CIDCA 8348 isolated from a dairy product present a great potential as probiotic for human or animal use and can be used also for producing functional foods.

## Figures and Tables

**Table 1:** Minimum inhibitory concentrations (MIC) for antibiotic resistance.

Antibiotics	MIC (mg L^−1^)						
	Breakpoints^a^	CIDCA 8321	CIDCA 8345	CIDCA 8348	CIDCA 83115	CIDCA 83111	CIDCA 83113
Ampicillin	**2**	<0.032	<0.032	<0.032	<0.032	<0.032	<0.032
Clindamycin	**1**	<0.032	<0.032	<0.032	<0.032	<0.032	<0.032
Chloramphenicol	**4**	**8**	**16**	2	2	1	2
Erythromycin	**1**	<0.125	<0.125	<0.125	<0.125	<0.125	<0.125
Gentamicin	**16**	<0.5	<0.5	<0.5	<0.5	<0.5	<0.5
Kanamycin	**32**	<2	<2	<2	<2	<2	<2
Streptomycin	**64**	<0.5	<0.5	<0.5	<0.5	<0.5	<0.5
Tetracycline	**8**	<0.125	<0.125	4	2	4	<0.125

^a^These are the recommended breakpoints for heterofermentative lactobacilli EFSA Panel on Additives and Products or Substances used in Animal Feed (2012) [20].

**Table 2:** Antimicrobial activity of *Lactobacillus kefiri* strains against pathogens by agar spot test.

Growth inhibition ability						
Strain	CIDCA 8321	CIDCA 8345	CIDCA 8348	CIDCA 83115	CIDCA 83111	CIDCA 83113
Gram negative bacilli						
*Pseudomona aeruginosa *	++	+	++	+	+	+
*Salmonella *Enteritidis	+	−	+	−	+	+
*Shigella flexneri *	+	−	+	−	+	−
*EHEC *	−	−	−	−	−	−

Gram positive bacilli						
*Listeria monocytogenes *	+	−	+	−	+	−
*Bacillus cereus *	++	+	++	+	+	++

Gram positive cocci						
*Enterococcus faecalis *	+	−	+	−	−	−
*Staphylococcus aureus *	++	+	++	+	++	+
